# Crotalphine Attenuates Pain and Neuroinflammation Induced by Experimental Autoimmune Encephalomyelitis in Mice

**DOI:** 10.3390/toxins13110827

**Published:** 2021-11-22

**Authors:** Aline C. Giardini, Bianca G. Evangelista, Morena B. Sant’Anna, Barbara B. Martins, Carmen L. P. Lancellotti, Adriano P. Ciena, Marucia Chacur, Rosana L. Pagano, Orlando G. Ribeiro, Vanessa O. Zambelli, Gisele Picolo

**Affiliations:** 1 Laboratory of Pain and Signaling, Butantan Institute, Sao Paulo 05503-900, SP, Brazil; aline.giardini@butantan.gov.br (A.C.G.); biah.evangelista31@gmail.com (B.G.E.); morena.santanna@butantan.gov.br (M.B.S.); barbara.martins@esib.butantan.gov.br (B.B.M.); vanessa.zambelli@butantan.gov.br (V.O.Z.); 2 Department of Pathological Sciences, Medical Science School Santa Casa of Sao Paulo, Sao Paulo 01221-020, SP, Brazil; luciapl@uol.com.br; 3 Laboratory of Morphology, Institute of Biosciences, São Paulo State University, Rio Claro 13506-52, SP, Brazil; adriano.ciena@unesp.br; 4 Laboratory of Functional Neuroanatomy of Pain, Instituto de Ciências Biomédicas, Universidade de Sao Paulo, Sao Paulo 05508-900, SP, Brazil; chacurm@icb.usp.br; 5 Laboratory of Neuroscience, Hospital Sírio-Libanês, Sao Paulo 01308-060, SP, Brazil; rosana.lpagano@hsl.org.br; 6 Laboratory of Immunogenetics, Butantan Institute, Sao Paulo 05503-900, SP, Brazil; orlando.ribeiro@butantan.gov.br

**Keywords:** neurodegenerative disease, neurodegeneration, inflammation, IL-17, glial cells

## Abstract

Multiple sclerosis (MS) is a demyelinating disease of inflammatory and autoimmune origin, which induces sensory and progressive motor impairments, including pain. Cells of the immune system actively participate in the pathogenesis and progression of MS by inducing neuroinflammation, tissue damage, and demyelination. Crotalphine (CRO), a structural analogue to a peptide firstly identified in *Crotalus durissus terrificus* snake venom, induces analgesia by endogenous opioid release and type 2 cannabinoid receptor (CB2) activation. Since CB2 activation downregulates neuroinflammation and ameliorates symptoms in mice models of MS, it was presently investigated whether CRO has a beneficial effect in the experimental autoimmune encephalomyelitis (EAE). CRO was administered on the 5th day after immunization, in a single dose, or five doses starting at the peak of disease. CRO partially reverted EAE-induced mechanical hyperalgesia and decreased the severity of the clinical signs. In addition, CRO decreases the inflammatory infiltrate and glial cells activation followed by TNF-α and IL-17 downregulation in the spinal cord. Peripherally, CRO recovers the EAE-induced impairment in myelin thickness in the sciatic nerve. Therefore, CRO interferes with central and peripheral neuroinflammation, opening perspectives to MS control.

## 1. Introduction

Multiple sclerosis (MS) is a chronic inflammatory and demyelinating disease which affects more than 2.8 million people worldwide [1]. The autoimmune inflammation that affects the central nervous system (CNS) progressively results in oligodendrocyte injury and demyelination. In the early stages, the axons are preserved, however, with the advance of the disease, the damages are irreversible [2]. The origin of MS is still unclear; however, the combination of environmental, such as lifestyle and viral exposure, and genetic factors may contribute to the development of the disease [3,4,5,6].

The literature shows multifocal inflamed regions in gray and white matter, with oligodendrocyte death and myelin sheath disruption [7,8]. The clinical presentation of MS is highly variable, making the diagnosis of the disease difficult [9]. The inflammatory response is mainly mediated by T helper (Th) cells, which play an essential role in the course of MS [10]. Pro-inflammatory cytokines such as interleukin (IL)-6, IL-17, IL-21, IL-22, IL-23, and tumor necrosis factor (TNF)-α are produced by Th17 cells [10,11,12,13]. IL-17, which is one of the main cytokines produced by these cells, acts as an inducer of neutrophil infiltration [13,14,15]. Th1 cells mediate CNS inflammation by stimulating macrophage infiltration and producing important cytokines including interferon (IFN)-γ, which plays a relevant role in spinal neuroinflammation [10]. In addition, B-lymphocytes play a critical role in tissue damage, propagating inflammation, and spinal demyelination in MS [16,17].

In addition to peripheral immune cells, activation of CNS resident cells also adds to the pathogenesis of MS, particularly microglia and astrocytes. Microglia cells are activated when tissue integrity is disturbed, secreting pro-inflammatory or anti-inflammatory cytokines, controlling the expression of anti-inflammatory molecules, regulating phagocytosis of debris and tissue repair, responses that depend on the changes that occurred in their specific microenvironment. Astrocytes strengthen and contribute to the maintenance of the integrity of the blood-brain barrier, restricting the entry of peripheral immune cells into the CNS [18] and modulating synaptic activity and plasticity [19,20]. These activated glial cells produce chemokines and cytokines, leading to the recruitment of additional immune cells to the CNS parenchyma besides producing exacerbated sensitization stages, including neural hypersensitivity [21]. Pain is one of the sensory alterations presented by MS patients, and can be of inflammatory, neuropathic or skeletal muscle origin [22]. It also affects the quality of life, aggravating symptoms such as depression, sleep disturbance, and mood [23,24,25,26,27]. Importantly, like many other progressive diseases, there is no specific treatment for MS, and therapies focus on delaying disease progression and promoting symptom relief, thus improving the patient’s quality of life.

Animal models have provided valuable answers regarding the mechanisms involved in the development and progression of MS and the efficacy of new drugs with therapeutic potential [11,28]. These experimental models reproduce CNS inflammation, demyelination of neurons, and some motor changes observed in MS [28,29,30,31], in addition to sensory alterations. Among the sensory alterations, the literature has demonstrated alterations in the pain threshold in both humans and animals that, in the MOG_35–55_-induced experimental autoimmune encephalomyelitis (EAE) model, arise before the onset of motor alterations [32,33,34], allowing for the study of pain previous to the first evidence of motor alterations, which would compromise pain sensitivity assessment.

Considering that MS is a chronic neurodegenerative disease whose treatment only delays progression and alleviates symptoms without interfering with the pathologic process, new therapeutic strategies are needed. Crotalphine (CRO) is a 14-amino acid peptide (EFSPENCQGESQPC) containing a disulfide bridge and a pyroglutamic acid [35], capable of inducing a potent and durable antinociceptive effect in acute and chronic pain models (including cancer pain and neuropathic pain) [35,36,37]. CRO induces antinociception by activating CB2 receptors, which, in turn, induces the release of endogenous peptides, particularly dynorphin A, the endogenous agonist of kappa opioid receptors [38]. It was also observed that CRO desensitizes transient receptor potential ankyrin ion channels (TRPA1) [39], a receptor that has a relevant role in the maintenance of inflammatory hyperalgesia [40]. Based on clinical studies showing the effectiveness of cannabinoids on clinical symptoms in patients with MS, particularly muscle stiffness and spasms, sleep disorders, and neuropathic pain [41,42], the CRO effect on clinical symptoms, neuroinflammation, and axonal demyelination of mice submitted to the MOG_35–55_-induced experimental autoimmune encephalomyelitis, an animal model of MS, is investigated here.

## 2. Results

### 2.1. CRO Caused Analgesia in EAE-Induced Pain and Attenuated Clinical Signs

**Figure 1:**
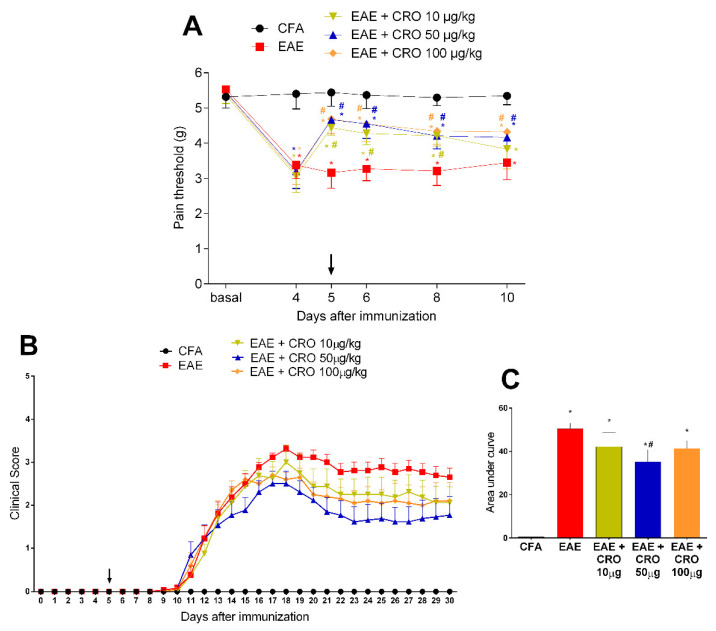
Evaluation of CRO effect in pain sensitivity and clinical signs of animals immunized with MOG_35–55_. Animals with EAE were treated with a single dose of CRO p.o. on the fifth day, in different doses. Pain sensitivity was measured by the electronic von Frey model (**A**) and motor impairment was evaluated according to a visual scale of clinical signs from 0 to 5 (**B**). For statistical analysis of clinical signs, the area under the curve was evaluated (**C**). Data represent mean ± SEM 8–10 animals per group. # *p* < 0.05 compared to untreated group (EAE). * *p* < 0.05 compared to the control group (CFA). The two-way ANOVA test, followed by Bonferroni post-hoc test was used in the electronic von Frey test, and the One-way ANOVA test was used, followed by Tukey’s post-hoc test, was used in the area under the curve.

CRO (10, 50, or 100 µg/Kg) or saline was first administered on the fifth day after immunization, in a single dose. The immunized animals (EAE) showed decreased nociceptive threshold (termed hypernociception) on the fourth day after immunization when compared to the complete Freund’s adjuvant (CFA) group (control). The hypernociception remained until the last day of evaluation. i.e., 10th day, when the assessment of the pain threshold was stopped due to the onset of motor dysfunctions. The treatment with CRO with doses of 10, 50, and 100 µg/kg, resulted in a partial reversal of the EAE-induced hyperalgesia (Figure 1A). The experiments were performed in the morning and the pain threshold was assessed 1 h after the treatment with CRO.

The first motor impairment symptom appeared on the 11th day after immunization and increased progressively, peaking around the 17–18th day. CRO, at the dose of 50 µg/kg, reduced the disease severity, represented by lower clinical scores, when compared to the untreated group (Figure 1B,C). This dose was chosen for subsequent experiments.

### 2.2. CRO Reverts EAE-Induced Motor Impairment

**Figure 2:**
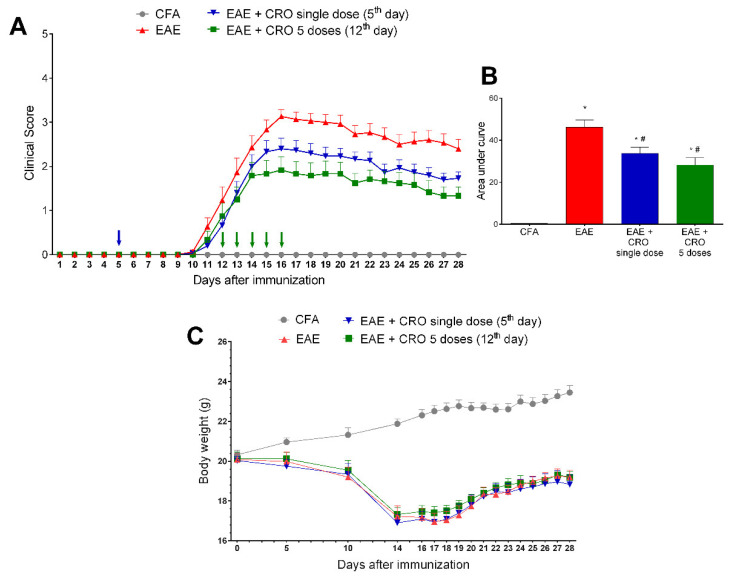
Evaluation of CRO effect on clinical signs and body weight of animals immunized with MOG_35–55_ and treated before or after motor impairment. Animals were immunized with MOG_35–55_ and treated with CRO in a single dose on the fifth day after immunization (blue arrow), or in five doses (green arrows, one daily dose for five consecutive days starting on the 12th day after immunization). Clinical signs were evaluated daily (**A**), and, for statistical analysis, the area under the curve was evaluated (**B**). Body weight (g) was measured during the course of the disease (**C**). Data represent mean ± SEM 8–10 animals per group. * *p* < 0.05 compared to the CFA group. # *p* < 0.05 compared to the untreated group (EAE). A one-way ANOVA test was used, followed by Tukey’s post-hoc test in the area under the curve.

To evaluate the efficacy of CRO after the onset of motor impairment, CRO was administered for five consecutive days (one dose by day), starting from the 12th day of immunization. The effectiveness of this protocol was compared with the single-dose protocol. The EAE groups showed a progressive increment in clinical score from the 11th day after immunization, peaking at the 16th day. Both groups treated with CRO showed lower clinical signs scores when compared to the untreated group, showing the potential of CRO to interfere with motor impairment even after its onset as well as to prevent it (Figure 2A,B). At this point, the analgesic effect of CRO was not evaluated, since it is not possible to apply the nociceptive test due to the motor impairment. The body weight of the animals was measured during the course of the disease and, as expected, EAE interfered with the weight gain of the animals (Figure 2C). Crotalphine did not alter the EAE-induced weight loss (Figure 2C).

### 2.3. CRO Reduced EAE-Induced Inflammatory Infiltrates in the Spinal Cord 28 Days after Immunization

**Figure 3:**
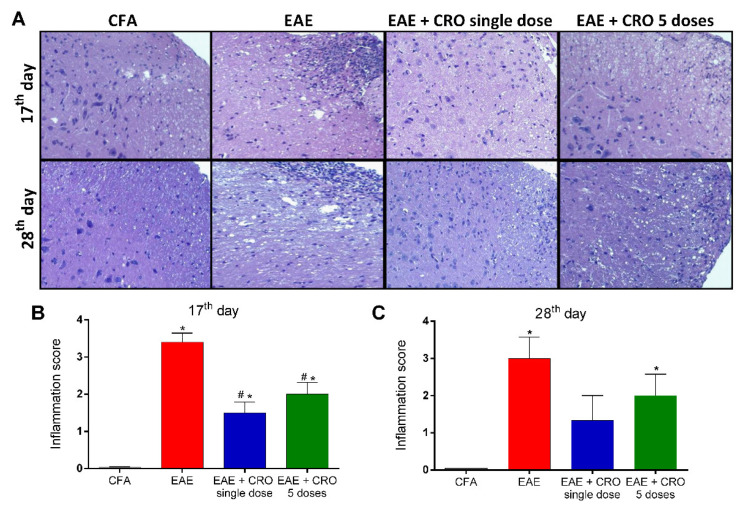
A single dose of CRO reduces cellular infiltration in the spinal cord. The animals were sedated and euthanized for spinal cord collection. Representative 20× microscopic fields of transverse sections of the spinal cord stained with hematoxylin and eosin ((**A**), panel). Quantification of the inflammatory score (0–4) in 17th (**B**) and 28th (**C**) days. Data represent the mean ± SEM of 3 animals per group. * *p* < 0.05 compared to the control group (CFA). # *p* < 0.05 compared to the untreated group (EAE). One-way ANOVA was used, followed by Tukey’s post-hoc test.

Central nervous system inflammation is a hallmark of MS pathogenesis [43]. Therefore, the effect of CRO in EAE-induced leukocyte migration to the spinal cord was investigated. The level of cellular infiltrate was scored from 0 (no cellular infiltrate) to 4 (high cellular infiltrate) As shown in panel (Figure 3A), HE staining revealed that there was no cell infiltration in the control group treated with CFA, and the spinal cord of EAE mice displayed mixed cellular infiltrate with numerous perivascular clusters. Of interest, treatment with CRO significantly reduces the EAE-induced cell infiltration at the peak of the disease (Figure 3B), with no difference observed on the 28th day after immunization (Figure 3C).

### 2.4. CRO Decreased EAE-Induced Glial Cells Immunoreactivity in the Spinal Cord

**Figure 4:**
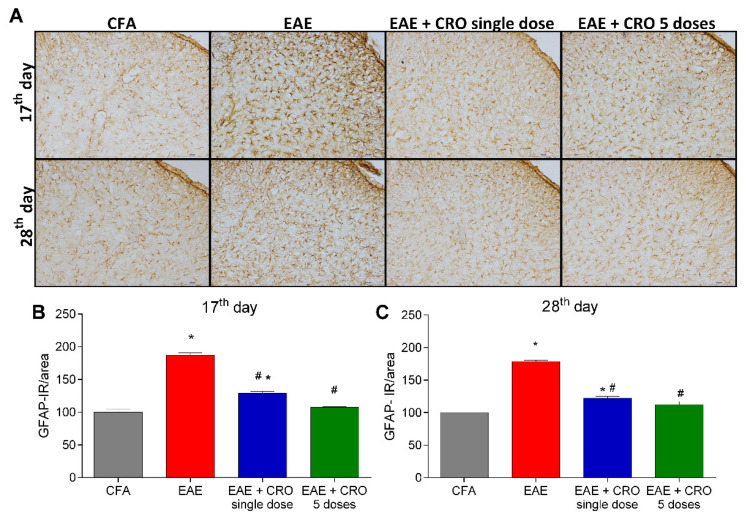
CRO effect on immunoreactivity for astrocytes in the spinal cord of EAE animals. Animals immunized with MOG_35–55_ and treated or untreated with CRO were perfused for the spinal cord collection. Representative 20× microscopic field of transverse sections of the spinal cord (L3-L6) stained with GFAP ((**A**), panel). Immunohistochemistry analysis on 17th (**B**) and 28th days (**C**) after immunization. Immunoreactivity (IR) was quantified using Image J software. Data represent mean ± SEM of 3 animals per group. # *p* < 0.05 compared to EAE group. * *p* < 0.05 compared to control group (CFA). A one-way ANOVA test was used, followed by Tukey’s post-hoc test.

**Figure 5:**
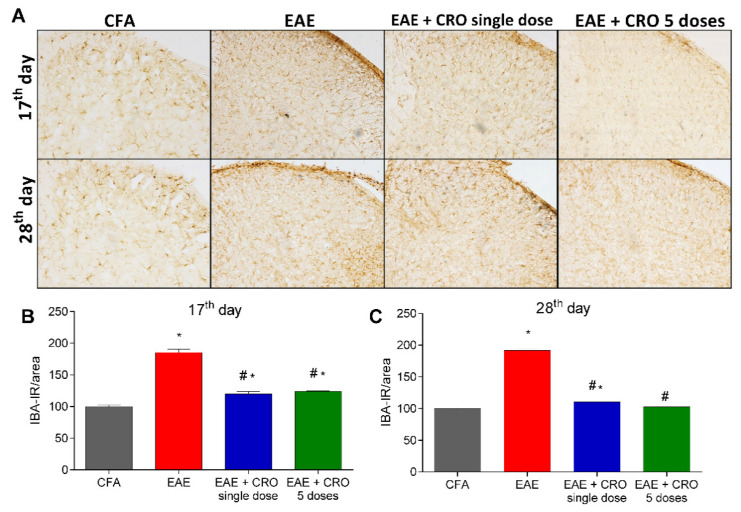
Evaluation of immunoreactivity for microglia in the spinal cord of animals with EAE and treated with CRO. Animals immunized with MOG_35–55_ and treated or untreated with CRO were perfused and had the spinal cords (L3-L6) collected. Representative 20× microscopic field of transverse sections of the spinal cord (L3-L6) stained with Iba-1 ((**A**), panel). Immunohistochemistry analysis on the 17th (**B**) and 28th (**C**) days after immunization. Immunoreactivity was quantified using Image J software. Data represent mean ± SEM of 3 animals per group. # *p* < 0.05 compared to EAE + sal group. * *p* < 0.05 compared to control group (CFA). A one-way ANOVA test was used, followed by Tukey’s post-hoc test.

Besides leucocyte infiltration, activation of resident CNS cells likewise contributes to the pathogenesis of EAE [44,45]. Next, the effect of CRO on EAE-induced astrocytes and microglia activation in the dorsal horn of the spinal cord was evaluated. Immunoreactivity for GFAP (glial fibrillary acidic protein), an astrocyte marker, and for Iba-1, a microglia marker was evaluated on the17th (clinical signs peak) and 28th (period of stabilization of clinical signs) days. The results show that EAE increases the reactivity for GFAP (Figure 4A,B) and Iba-1 (ionized calcium-binding adaptor-1) (Figure 5A,B) when compared with the control group (CFA), in both periods. On the other hand, the single-dose and five doses CRO protocol revert astrocyte and microglia activation induced by EAE (Figure 4 and Figure 5).

### 2.5. CRO Mitigate EAE-Induced IL-17 and TNF-α Release in the Spinal Cord at the Peak of the Disease

**Figure 6:**
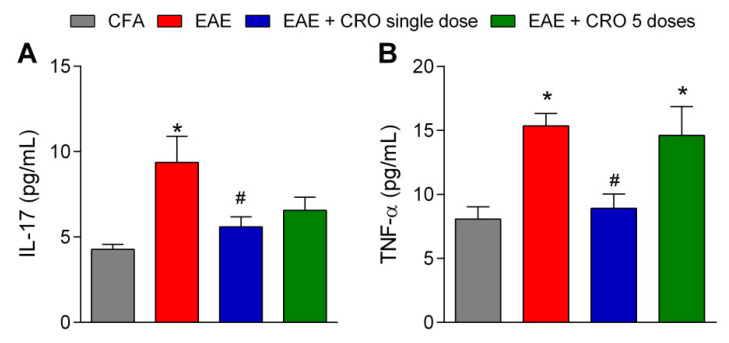
CRO effect on cytokine release in the spinal cord of EAE animals. Evaluation of the expression of IL-17 (**A**) and TNF-α (**B**) cytokines was analyzed by multiplex assay. The animals were immunized, euthanized, and the lumbar portion of the spinal cord was collected on the 17th day after immunization. Data represent the mean ± SEM of 5–7 animals per group. * *p* < 0.05 compared to the CFA group. # *p* < 0.05 compared to the untreated group (EAE). One-way ANOVA was used, followed by Tukey’s post-hoc test.

IL-17 and TNF-α are proinflammatory cytokines that activate T cells and other immune cells to produce and release a variety of cytokines and chemokines, and to express cell adhesion molecules [46]. Figure 6 shows that the levels of IL-17 and TNF-α are increased in the spinal cord of EAE animals at the peak of disease. Of interest, the treatment with CRO on the 5th day prevents EAE-induced IL-17 and TNF-α release. Moreover, the protocol of five doses of CRO does not prevent IL-17 and TNF-α release (Figure 6).

### 2.6. CRO Not Interfered with the Nerve Growth Factor Expression in the Spinal Cord of Animals with EAE

**Figure 7:**
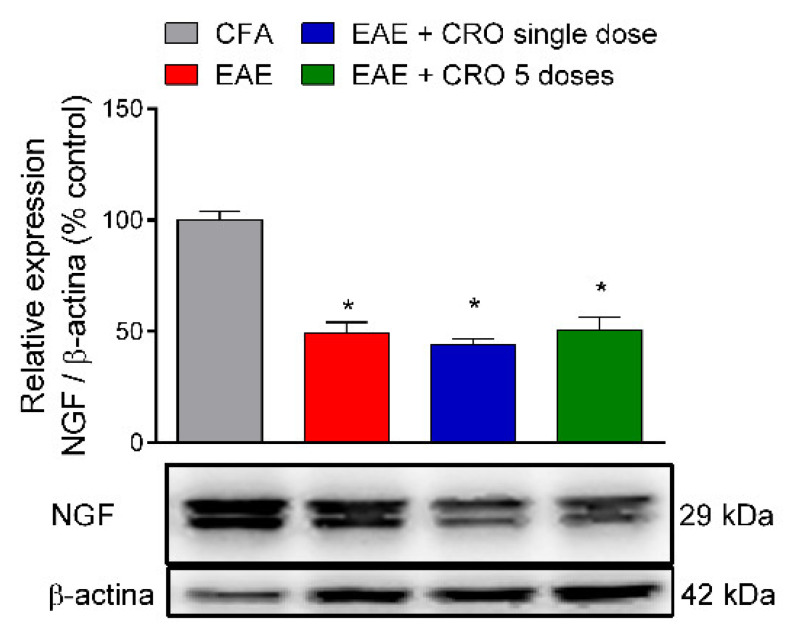
Effect of CRO in neurotrophic factor in the spinal cord of animals immunized with MOG_35–55_. Animals, on the 28th day after immunization, were sedated and euthanized for the spinal cord collection. The expression of neural growth factor (NGF) was evaluated by western blotting. Beta-actin was used as the loading control. Results were normalized by CFA group expression. Results are expressed as the mean ± SEM of 5 animals per gropu. * *p* < 0.05 in relation to the CFA group. One-way ANOVA followed by Tukey’s post-hoc test.

Nerve growth factor (NGF) is a neurotrophin associated with the differentiation and survival of numerous neurons localized in the peripheral and CNS [47]. It was next investigated if CRO could interfere with the expression of NGF in the lumbar section of the spinal cord (L3–L6) on the 28th day after immunization. Our data confirm the results of the literature showing that NGF expression is reduced in the spinal cord of EAE mice; however, CRO does not change those levels (Figure 7).

### 2.7. CRO Prevented the Peripheral Demyelination of the Sciatic Nerve Induced by EAE

We have previously demonstrated that EAE decreases sciatic nerve myelin thickness when quantified through the g-ratio estimative (i.e., dividing axon diameter by the fiber diameter) [48]. Here, we checked whether CRO would have a beneficial effect on EAE-induced demyelination.

**Figure 8:**
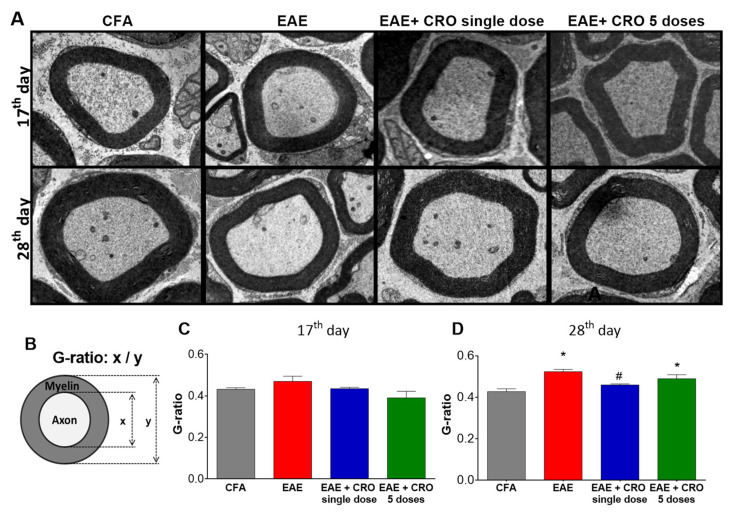
Effect of CRO on the peripheral nerve myelin sheath of animals with EAE. Animals were sedated, euthanized, perfused and the sciatic nerve was collected for evaluation by transmission electron microscopy, 7500× magnification. Representative microscopic fields of transverse sections of the peripheral nerve stained with toluidine blue solution ((**A**), panel). The G-ratio (**B**) was measured using ImageJ software for evaluation of axonal myelin content on the 17th (**C**) and 28th days (**D**) after immunization. Data represent the mean ± SEM of 3 animals per group. * *p* < 0.05 compared to the control group (CFA). # *p* < 0.05 compared to the untreated group (EAE). One-way ANOVA was used, followed by Tukey’s post-hoc test.

In order to address this question, the distal portion of the sciatic nerve was collected on the 17th and the 28th days after immunization and its morphology was analyzed (Figure 8A) by transmission electron microscopy. The results demonstrated the presence of intact fibers, with a similar distribution of small and large diameter myelinic fibers, non-myelinic fibers, and Schwann cell nuclei in the control group (CFA). At the peak of the disease (17th day after immunization), EAE animals showed no reduction in myelin sheath thickness (Figure 8C). However, on the 28th day after immunization (Figure 8D), a decrease in the sciatic nerve myelin thickness, defined by an increase in the g-ratio when compared with control animals, was detected. Importantly, animals treated with a single dose of CRO on the fifth day after immunization have a thickness of myelin sheaths similar to the control group. This data shows that CRO prevents EAE-induced demyelination, which may preserve the nerve conduction and contribute to the analgesic effect.

## 3. Discussion

MS is a chronic inflammatory disease of autoimmune origin which, demyelinates neurons from the CNS. Components of the CNS are recognized as antigens, more specifically the myelin sheath of the axons. T lymphocyte-mediated CNS aggression is observed in MS [49], as well as in the activation of glial cells (microglia and astrocytes) in both the spinal cord and brain regions [50,51], innate immune cell activation, and cytokines and chemokines release [52]. This inflammatory response greatly alters the properties of neurons, leading to demyelination and axonal loss [53], as well as to several motor, cognitive and sensory alterations [54,55].

Presently, the effect of CRO on disease development was evaluated. CRO is an analgesic peptide, firstly identified in the venom of *Crotalus durissus terrificus* snake [35], which induces long-lasting antinociceptive effect in animals, observed in acute and chronic pain models, which do not induce some of the side effects observed for analgesic drugs, such as alteration in spontaneous motor and general activity or the development of tolerance to its antinociceptive effect after prolonged treatment [35,36,37]. It was previously demonstrated by our group that CRO induces analgesia mediated by the activation of CB2 cannabinoid receptors [38]. First, we evaluated the disease development regarding motor impairment through the observation of clinical signs. The results showing that CRO ameliorates the EAE-induced clinical signs corroborate data from the literature, where the administration of a phytocannabinoid, an agonist of CB2 receptors, attenuated the clinical severity of the disease in mice, both preventively and after the onset of clinical signs [56].

In addition to the motor impairment, central neuropathic pain is observed in animal models of EAE, occurring due to prolonged inflammation in the spinal cord, resulting in activation of glial cells and aggression to the myelin sheath, causing painful hypersensitivity [31,57]. Thus, activation of spinal astrocytes and microglia seems to be a key element in the generation and maintenance of peripheral neuropathic pain [58,59], reducing the nociceptive threshold of animals before neurological dysfunction occurs [60]. It was presently shown that CRO partially reverses EAE-induced mechanical hypersensitivity. Importantly, the analgesic effect was already observed 1 h after its administration. Considering the rapid analgesic effect observed, as well as the fact that hypernociception is detected before the onset of clinical signs, it is assumed that the observed pain does not depend on the onset of central inflammation and that the observed analgesic effect is due to mechanisms independent of the anti-inflammatory observed effect.

As previously pointed out, central inflammation is a key factor in the progressing of EAE as well as MS [53,54,55]. Our data clearly demonstrated that crotalphine is capable of preventing the cell influx to the CNS. However, in addition to migrated cells, resident cells also play an important role in the onset of disease development. Microglial reactivity is manifested by morphological changes, modifications in the expression of surface molecules, and secretion of several substances such as cytokines, trophic factors, and chemokines [61,62,63]. Specifically, in the acute period of EAE, microglia reactivity was detected along with increased clinical symptomatology. Treatment with a microglial inhibitor or microglia depletion has been shown to induce beneficial effects on EAE symptoms, demonstrating that microglial cells play a role in the pathogenesis of EAE [64,65]. In addition, it has been reported that activated microglia may release a large variety of molecules, which may contribute to immune cell recruitment and the spread of inflammatory response [64,65,66,67]. Astrocytes also play important roles in the development of chronic pain, producing neurotoxic mediators, cytokines, and chemokines, with proinflammatory activity [21,68,69]. Here, the literature findings were reproduced, showing that EAE increases microglial and astrocytes labeling, both at the peak of the disease and in the late period [31,61]. Importantly, CRO decreased astrocytes and microglia reactivity in both periods, observed in the dorsal horn of the spinal cord, a region related to the nociceptive pathway [70]. In addition to that, histological analysis of the spinal cord showed that CRO decreases cell infiltrates in EAE mice, suggesting a correlation with reduced glial activation, TNF-α cytokine production, and lower clinical signs. These results point to the reduction of the neuroinflammation induced by CRO as a key factor for the antinociceptive effect and the improvement of clinical signs.

The contribution of Th17 cells and its effector cytokine signature, IL-17, is well described as promoters of the induction and progression of MS/EAE [71,72,73]. The role of Th1 cells in the process is known, but Th17 cells have a greater proliferative capacity than Th1 cells, in addition to being able to cross the blood-brain barrier (BBB) more easily [71]. Here, it was confirmed that IL-17 levels are up-regulated in the EAE group. Of interest, CRO prevented the IL-17 increase in the spinal cord (17th day). It is evident that IL-17 is a key cytokine in the EAE model [74]. In fact, anti-IL17A treatment showed satisfactory results in attenuating the development of EAE [75], for example, in reducing the clinical signs and improving the histological findings. In addition, IL-17 knockout mice exhibited delayed onset, reduced clinical scores, and early recovery after immunization [76]. Data from our group also demonstrated the importance of the IL-17 to the development of EAE using crotoxin, the main neurotoxin from the venom of the *Crotalus durissus terrificus* snake. In these studies, crotoxin attenuated clinical signs by inhibiting the release of IL-17 and reducing CD4^+^IL-17^+^ cell proliferation in lymph nodes [77]. These data point out that the reduction of this pro-inflammatory cytokine is one of the main factors that contribute to CRO attenuation of disease progression. Importantly, although the central release of IL-17 was decreased, the proliferation of Th17 cells in the lymph nodes, at the onset of the immune process, was not altered by CRO (data not shown). In addition to IL-17, the profile of TNF-α was also investigated, since IL-17 and TNF-α are the two major cytokines involved in MS [78,79,80]. Our results demonstrated that a single dose of CRO reversed EAE-induced TNF-α release in the spinal cord at the peak of the disease. The contribution of TNF-α to the development and progression of MS is well established. However, despite the pro-inflammatory actions of TNF-α, the use of anti-TNF-α monoclonal antibodies in MS patients, in addition to being ineffective, promotes an unexpected worsening of the disease [81], indicating that this cytokine also plays a protective role. Several studies have shown that the pathogenic and homeostatic activities of TNF-α are mediated by distinct cellular and molecular pathways and depend on the type of TNF-α receptor that is activated. Astrocytes, oligodendrocytes, and Treg cells express the TNFR2 receptor; the activation of this receptor mediates neuronal survival, re-myelination, and acts on immunoregulation. On the other hand, activation of TNFR1 receptors induces neuroinflammation and demyelination. Substances that activate CB2 type cannabinoid receptors act on glial cells and neurons, inhibiting TNF-α release and having antioxidant action [82]. Thus, the decrease in TNF-α levels presently observed may be a consequence of CB2 receptor activation, and since this decrease is partial, it is plausible that the remaining TNF-α would continue to exert its neuroprotective effect.

The increased chemokines levels detected in EAE makes the BBB more permeable to inflammatory cells; thus peripheral macrophages infiltrate the CNS [83]. Microglia and macrophages are considered important in the development of EAE and actively contribute to the pathogenesis and progression of the disease [84], causing the release of proinflammatory cytokines and leading to gliosis, inflammation tissue damage, and demyelination, culminating in neuronal death (neurodegeneration) in the CNS [61,85,86]. During the acute stage of EAE, NGF and its tyrosine kinase receptor (TrkA) expression are decreased in the CNS in rats [87]. Furthermore, studies suggest that NGF is responsible for the induction of axonal regeneration, survival, maintenance, and the differentiation of oligodendrocytes, as well as for facilitating the migration and proliferation of oligodendrocyte precursors to the myelin injury sites, a key role in the recovery of demyelinating diseases and stimulating recovery from neuroinflammatory diseases, including EAE [88,89,90]. On the 28th day after immunization, the expression of neurotrophin NGF was analyzed. In our study, corroborating data from the literature, a reduction of NGF in the spinal cord of the EAE animals was observed; however, it was not prevented by CRO.

It is noteworthy that MS is a disease that affects the CNS and that the literature is scarce regarding the effects of EAE on the demyelination of peripheral nerves. To verify possible peripheral demyelination in this animal model of MS and whether the treatment with CRO can interfere in this process, the morphology of the sciatic nerve was analyzed by transmission electron microscopy. This methodology has been applied for the evaluation of peripheral neurodegeneration, such as demyelination and the effect of neuroprotective therapies on these processes [91]. It was presently observed that this autoimmune disease alters the structure of the sciatic nerve myelin sheath of animals. Our results, together with results previously published [92], indicate that sciatic nerve demyelination may contribute to the neuropathic pain detected in this EAE model. In contrast to previous knowledge about the expression of myelin basic protein (MBP) and MOG exclusively at the CNS, it is currently known that these proteins are expressed in the peripheral nervous system and can therefore be attacked by autoantibodies, contributing to peripheral demyelination [93,94,95,96]. Interestingly, treatment with a single dose of CRO induces improvement in myelin fiber thickness. These results are promising and indicate that CRO may prevent demyelination or promote remyelination. Further studies to confirm these hypotheses are necessary.

## 4. Conclusions

Our results demonstrated that CRO interferes with the normal course of EAE, acting on both central and peripheral sites. This process occurs through the inhibition of CRO with cytokines release and reduction in the activation of glial cells and inflammatory infiltrate, indicating lower neuroinflammation and central sensitization, thus attenuating hyperalgesia and clinical signs. These results show the effectiveness of CRO in this animal model of neurodegenerative disease, indicating important central sites to be controlled in order to interfere with disease development.

## 5. Materials and Methods

### 5.1. Animals

Female C57BL/6J mice were used, supplied by Butantan Institute Central Animal Facility. The animals were kept with water and feed ad libitum in an appropriate soundproof room, controlled temperature (22 °C ± 1), and light-dark cycle (12/12 h), with a maximum of 6 animals per cage. All procedures were performed according to the “Ethical Guide for the Use of Pain-Sensitive Animals in Pain Testing”, published by IASP [97], reported following the Animal Research Reporting of In Vivo Experiments (ARRIVE) guidelines [http://www.nc3rs.org.uk/arrive-guidelines (accessed on 8 September 2021)] and approved by the Ethics Committee on Animal Use of the Butantan Institute (CEUAIB protocol number 7334170718).

### 5.2. Experimental Design

**Figure 9:**
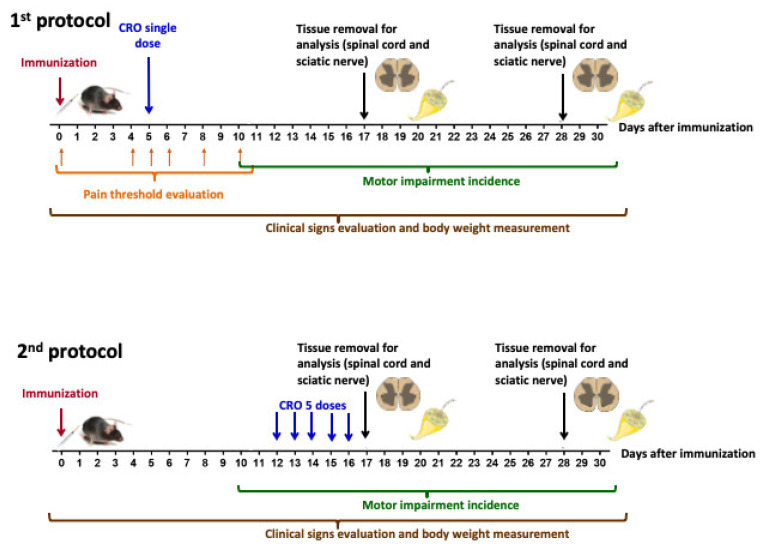
Experimental design for the evaluation of CRO effect on EAE development.

Mice were handled according to Figure 9. Briefly, EAE was induced in female C57BL/6J mice. Pain sensitivity was evaluated before (day 0) and on the fourth, fifth, sixth, eighth^,^ and 10th days (as Section 5.4) after immunization (as Section 5.3). Clinical signs were daily evaluated. CRO was administered in a single dose (on the 5th day after immunization–1st protocol) or in five repeated doses from the 12th day (one daily dose for five consecutive days–2nd protocol). At the 17th (peak of disease) and 28th (remission phase) days after immunization, the spinal cord was collected for inflammatory infiltrate determination (as Section 5.6), glial cells immunoreactivity evaluation (as Section 5.7), cytokines release measurement (as Section 5.8), and nerve growth factor expression (as Section 5.9). At the same periods, the sciatic nerve was removed for myelin thickness determination (as Section 5.10).

### 5.3. Induction of the EAE

The EAE model was induced as previously described [98]. Animals were anesthetized (isoflurane 1.5–2% in oxygen) and immunized subcutaneously at the base of the tail (s.c.) with 200 μg of myelin oligodendrocyte glycoprotein (MOG_35–55_) peptide (Proteimax Technology, Sao Paulo, SP, Brazil) emulsified in incomplete Freund’s adjuvant (IFA) supplemented with *Mycobacterium tuberculosis* (4 mg/mL, H37Ra, Difco™, Detroit, MI, USA). Intraperitoneal (i.p.) injection of 300 ng of pertussis toxin (Sigma-Aldrich™, St. Louis, MO, USA) was performed immediately after immunization and 48 h later. Control animals were treated with the vehicle (complete Freund’s adjuvant—CFA, s.c. and pertussis toxin, i.p.). Animals were daily monitored according to the following clinical scores: 0.0, no symptoms; 0.5, some loss of tail tone; 1.0, loss of tail tone; 1.5, hip weakness upon ambulation; 2.0, hip weakness and partial hind limb paresis; 2.5, partial hind limb paralysis; 3.0, total hind limb paralysis but still moving on the cage; 3.5, complete hind limb paralysis and forelimbs weakness; 4.0, total hind limb paralysis and partial forelimbs paralysis; 4.5, complete hind limb paralysis, partial paralysis of the forelimbs, decreased responsiveness (consider euthanasia); 5, Immobile and unresponsive, moribund (immediate euthanasia). In the occurrence of score 3, food and water were turned available inside the cage. In the occurrence of score 4, animals were evaluated twice a day and if this score persists for three consecutive assessments, euthanasia was applied and the score of 5 was considered to the end of the period of evaluation. Body weight was measured during the course of the disease.

### 5.4. Evaluation of Pain Sensitivity: Determination of Mechanical Hyperalgesia by Electronic von Frey Test

The animals were habituated in the experimental room 30 min before the measurements. The mice were individually placed in bottomless acrylic boxes. On the bottom of the boxes, a wire mesh allows access to the plantar surface of the hind paw of the animal. A polypropylene disposable tip of 0.5 mm^2^ with increasing perpendicular force was applied on the plantar surface of the hind limb until the animal shows the paw withdrawal reaction, thus indicating its nociceptive threshold [99]. The tip is coupled to a transducer, which indicates the value in grams of the force applied to the animal’s paw; the accuracy of the device is 0.1 g and the device records the maximum force applied (Insight Equipamentos Ltda., Ribeirao Preto, SP, Brazil). Pain sensitivity was evaluated before (baseline) and at different periods after immunization with MOG_35–55_, before they presented motor alterations.

### 5.5. Pharmacological Treatment with CRO

Crotalphine (<E-F-S-P-E-N-C-Q-G-E-S-Q-P-C, where <E is pyroglutamic acid and 7C-14C forms a disulfide bond; MW 1534.6 Da) was synthesized by Proteimax Biotecnologia, Brazil (São Paulo, SP, Brazil) as previously described [35], and was administered by oral route, diluted in sterile saline (NaCl 0.9% in distilled water). For dose setting of CRO, animals with EAE were allocated into different groups which received CRO (10, 50 or 100 μg/kg, p.o.) or saline (p.o.), on the fifth day after immunization (1 day after the onset of nociceptive response, the first symptom of the disease). Administration of CRO was based on previous assays showing long-lasting analgesic effects in experimental models of pain [36,37]. CRO (50 μg/kg) was also administered in five repeated doses (one daily dose for five consecutive days) from the 12th day (one day after motor impairment manifestation). The protocol of five consecutive doses was based on previous results demonstrating the greatest difficulty in controlling the symptoms of the disease after the onset of clinical signs [77]; however, it was performed to determine whether crotalphine would be able to interfere with the development of the disease even after the motor impairment trigger.

### 5.6. Histology of Spinal Cord to Inflammatory Infiltrate Determination (Hematoxylin and Eosin Stain)

To characterize the cellular infiltrate in the spinal cord, histological analysis was performed in EAE animals after CRO or saline treatment. The region comprised between lumbar segments from the spinal cord was removed and fixed in 10% of paraformaldehyde (PFA) solution for 24 h, at room temperature. After the fixation period, the tissues were transferred for a 70% ethyl alcohol solution, dehydrated, embedded in paraffin, and cut into sections of 5 µm thickness. The sections were stained with hematoxylin-eosin (HE). This classical technique in histological diagnosis has two dyes: hematoxylin, which stains all the nuclei of all cells, and eosin, which stains the cytoplasm of all cells [100].

### 5.7. Glial Cells Immunoreactivity Evaluated by Immunohistochemistry Assay

At the 17th (peak of disease) and 28th (remission phase) days after immunization, animals were anesthetized using ketamine (75 mg/kg i.p.) and xylazine (10 mg/kg, i.p.) and were transcardially perfused with 0.9% saline followed by 4% of PFA solution. The spinal cord was collected, and tissues were included in PFA solution for 4 h to be post-fixed. After that, tissues were kept, for at least 48 h, in a 30% sucrose solution in a refrigerator (2–8 °C). After this period, the regions of the spinal cord corresponding to the L3-L6 were sectioned (30 μm) in a microtome, collected in an anti-freezing solution (500 mL of phosphate buffer 0.05 M, 300 mL of ethylene glycol, and 150 g of sucrose) and kept at −20 °C. After washing three times with phosphate buffer (PB 0.1M, pH 7.4), the material was incubated free-floating for 12–16 h with specific Iba-1 (ionized calcium-binding adaptor-1, 1:2000, Wako Chemicals, Richmond, VA, USA) and GFAP (glial fibrillary acidic protein, 1:1000, Sigma-Aldrich™, St. Louis, MO, USA) antibodies in PB containing 0.3% Triton X-100 and 5% normal donkey serum. After that, sections were washed in PB (3 times), incubated with the biotinylated secondary antibody (anti-rabbit, 1:200 in PB containing 0.3% Triton X-100) for 2 h, washed 3 times in PB, and incubated with the avidin complex -biotin peroxidase (ABC Elite, Vector) for 2 h. After the washing process, tissues were reacted in diaminobenzidine (DAB, Sigma Aldrich™, USA) and 0.01% hydrogen peroxide in PB. The slices were mounted on gelatinized slides and kept at 37 °C for 48 h, dehydrated at room temperature, cleaned, and covered with a coverslip. Immunoreactivity quantification was performed in the dorsal horn of the spinal cord using ImageJ software (NIH/EUA).

### 5.8. Evaluation of IL-17 and TNF-α Release by Multiplex Assay

To investigate the IL-17 and TNF-α cytokines release, EAE animals treated with CRO or saline were euthanized, and the lumbar portion of the spinal cord (L3-L6) was collected on the 17th day after immunization. Collected tissue was homogenized in RIPA buffer (Sigma-Aldrich™, USA) containing protease inhibitors cocktail (1:100, Sigma-Aldrich™, USA) and phosphatase (1:300, Sigma-Aldrich™, USA). The homogenate was centrifuged for 5 min at 10,000× *g* and 4 °C. An aliquot of the supernatant was used for protein determination by the Bradford method [39,101]. The samples were normalized (3 µg/µL) and the protein concentration was determined using the commercial kit (Millipore, Burlington, MA, USA) by xMap method (MULTIPLEX), All the samples were done in duplicate and read using the Luminex 200 equipment—xPonent software version 4.2 (LEAC lab, Sao Paulo, SP, Brazil).

### 5.9. Nerve Growth Factor Expression by Western Blotting Assay

On the 17th day after immunization, animals were euthanized under anesthesia and the L3-L6 regions of the spinal cord were removed. The samples were homogenized in buffer containing Hepes-NaOH (1M, pH 7.9), EGTA (200 mM), NaCl (0.9%), Triton-X 100 (1%), and phosphatase and protease inhibitor (1:300, Sigma-Aldrich™, USA). The samples were centrifuged at 12,000× *g* rpm for 20 min at 4 °C. After centrifugation, an aliquot of the supernatant was used for protein concentration determination using the method of Bradford (Thermo Fisher Scientific, Waltham, MA, USA) [101]. Aliquots containing 30 µg of protein were boiled in Laemmli buffer 5× and after that subjected to polyacrylamide gel electrophoresis (SDS-PAGE), in an Mini-Protean apparatus for mini gel (BioRad, Hercules, CA, USA). After separation by electrophoresis, the proteins were transferred to a nitrocellulose membrane (BioRad, Hercules, CA, USA), and subsequently, the membrane was blocked in TBST (20 mM Tris-HCl, 150 mM NaCl, and 0.1% Tween 20) containing 5% of BSA for 1 h, then washed in TBST, followed by incubation with antibody NGF (1:1000, Abcam, Boston, MA, USA) overnight at 4 °C. Then, membranes were incubated with anti-IgG from the animal producing the respective peroxidase-conjugated primary antibody (1:5000, Abcam, USA), for 2 h at room temperature. ECL (Pierce) solution with a digital image capture system (UVITEC Cambridge) was used for bands visualization and the UVITEC Cambridge program was used for optical density determination. Results were normalized by membrane incubation with beta-actin (1:5000, Sigma Aldrich™, USA).

### 5.10. Transmission Electronic Microscopy

After intraperitoneal anesthesia with urethane (3 g/kg), animals were perfused with modified Karnovsky fixative solution containing 2.5% glutaraldehyde and 2% PFA in 0.1M sodium phosphate buffer solution (pH 7.4) [102]. After dissection of the muscles of the posterior thigh, the sciatic nerve was collected. The samples were post-fixed in 1% osmium tetroxide solution at 4 °C and then immersed in 5% aqueous uranyl acetate solution at room temperature. After that, the samples were dehydrated using a series of alcohols, then immersed in propylene oxide, and finally included in Spurr resin. Semi-thin sections (15 μM) were cut using Reichert Ultra Cut^®^ ultra-microtome (Leica, Wetzlar, Germany) and stained with 1% toluidine blue solution. Subsequently, the 60 nm ultrathin sections were collected on 200 mesh copper grids (Sigma-Aldrich™, USA) and contrasted with 4% uranyl acetate solution and 0.4% aqueous lead citrate solution [103]. The grids were examined using the Jeol 1010 transmission electron microscope (NEP/MEPA/ESALQ) at the University of São Paulo.

### 5.11. Statistical Analysis

Statistical analysis was performed using GraphPad Prism version 6 software (GraphPad, San Diego, CA, USA). Test one-way analysis of variance (ANOVA) followed by Tukey’s post-test was used for comparisons among three or more groups. Two-way analysis of variance (ANOVA) followed by Bonferroni post-test was used to compare repeated measures of the electronic von Frey test. The data are expressed as the mean ± SEM and *p* < 0.05 was indicative of a significant statistical difference.

## Data Availability

Not applicable.
